# PKM2-Induced the Phosphorylation of Histone H3 Contributes to EGF-Mediated PD-L1 Transcription in HCC

**DOI:** 10.3389/fphar.2020.577108

**Published:** 2020-11-25

**Authors:** Xiao Wang, Chao Liang, Xin Yao, Ruo-Han Yang, Zhan-Sheng Zhang, Fan-Ye Liu, Wen-Qi Li, Shu-Hua Pei, Jing Ma, Song-Qiang Xie, Dong Fang

**Affiliations:** ^1^ Institute for Innovative Drug Design and Evaluation, School of Pharmacy, Henan University, Kaifeng, China; ^2^ Institute of Chemical Biology, School of Pharmacy, Henan University, Kaifeng, China

**Keywords:** epidermal growth factor, Pyruvate kinase isoform M2, histone H3, programmed death-ligand-1, hepatocellular carcinoma

## Abstract

High expression of programmed death-ligand-1 (PD-L1) in hepatocellular carcinoma (HCC) cells usually inhibits the proliferation and functions of T cells, leading to immune suppression in tumor microenvironment. However, very little has been described regarding the mechanism of PD-L1 overexpression in HCC cells. In the present study, we found epidermal growth factor (EGF) stimulation promoted the expression of PD-L1 mRNA and protein in HCC cells. Inhibition of epidermal growth factor receptor (EGFR) could reverse EGF-induced the expression of PD-L1 mRNA and protein. Subsequently, we also observed that the phosphorylation level of Pyruvate kinase isoform M2 (PKM2) at Ser^37^ site was also increased in response to EGF stimulation. Expression of a phosphorylation-mimic PKM2 S37D mutant stimulated PD-L1 expression as well as H3-Thr^11^ phosphorylation in HCC cells, while inhibition of PKM2 significantly blocked EGF-induced PD-L1 expression and H3-Thr^11^ phosphorylation. Furthermore, mutation of Thr^11^ of histone H3 into alanine abrogated EGF-induced mRNA and protein expression of PD-L1, Chromatin immunoprecipitation (ChIP) assay also suggested that EGF treatment resulted in enhanced H3-Thr^11^ phosphorylation at the PD-L1 promoter. In a diethylnitrosamine (DEN)-induced rat model of HCC, we found that the expression of phosphorylated EGFR, PKM2 nuclear expression, H3-Thr^11^ phosphorylation as well as PD-L1 mRNA and protein was higher in the livers than that in normal rat livers. Taken together, our study suggested that PKM2-dependent histone H3-Thr^11^ phosphorylation was crucial for EGF-induced PD-L1 expression at transcriptional level in HCC. These findings may provide an alternative target for the treatment of hepatocellular carcinoma.

## Introduction

Hepatocellular carcinoma (HCC) is one of the most common cancers worldwide with a high mortality rate (Du et al., 2016; Nie et al., 2018). Immune escape is emerging as important contributors to the pathogenesis of HCC through remodeling the tumor microenvironment (Liu et al., 2018a; Mantovani et al., 2020). Aberrant overexpression of programmed death-ligand-1 (PD-L1) has been observed in hepatocellular carcinoma cells (Zhong et al., 2017). Unfortunately, PD-L1 expression by cancer cells usually inhibits the proliferation and functions of T cells in tumoral microenvironment, leading to immune suppression and impressive antitumor effects (Langhans et al., 2019; Liao et al., 2019). However, the mechanism of PD-L1 upregulation in HCC remains largely unclear.

The epidermal growth factor receptor (EGFR) belongs to the ErbB family of receptor tyrosine kinases (RTKs) (Patel and Leung, 2012; Maennling et al., 2019). Hyperactivation of EGFR in HCC is suggested to be associated with aggressive tumors, metastasis, and poor patient survival (Berasain and Avila, 2014; Lanaya et al., 2014). Numerous studies have showed that EGF/EGFR signal could increase PD-L1 expressions in non-small-cell lung cancer, colon cancer stem cells, renal cancer cells and glioblastoma (Li et al., 2018b; Chen et al., 2019b; Ma et al., 2020; Su et al., 2020). Therefore, this raise the possibility that EGF/EGFR signal is also attributable to the upregulation of PD-L1 in HCC.

Pyruvate kinase isoform M2 (PKM2) is a rate-limiting enzyme in glycolysis that plays a key role in tumor metabolism (Xie et al., 2016; Liu et al., 2017). Interestingly, under EGF stimulation, PKM2 can translocate to the nucleus and act as a transcriptional co-activator of many target genes associated with tumor cell growth and metastasis (Yang et al., 2011; Chen et al., 2019c). For example, PKM2 could translocate to nucleus and phosphorylate histone H3 at Thr^11^ residue in response to the activation of EGFR, leading to the expression of CCND1 and MYC at transcriptional level (Yang et al., 2012a). Notably, recent findings showed that PKM2 was closely positively related to PD-L1 expression in lung adenocarcinoma and colon carcinoma (Palsson-McDermott et al., 2017; Guo et al., 2019), while knockdown of PKM2 substantially inhibited PD-L1 expression in lung cancer cells (Guo et al., 2019). Although the expression of PKM2 was higher in HCC tissues than in adjacent tissues (Lv et al., 2018), it is still unknown whether PKM2 was involved in regulating PD-L1 expression in HCC. In the present study, we found that PKM2-induced phosphorylation of histone H3 is required for EGFR-mediated PD-L1 transcription in hepatocellular carcinoma.

## Materials and Methods

### Materials

All the inhibitors used in the present study were purchased from Cell Signaling Technology (Beverly, MA, USA). Rabbit monoclonal anti-PD-L1 antibody (ab205921), rabbit monoclonal anti-PKM2 antibody (ab206130), rabbit polyclonal anti-Histone H3 antibody (ab1791), rabbit polyclonal anti-Histone H3 (phosphor-Thr^11^) antibody (ab5168) were obtained from Abcam (Cambridge, MA, USA). Rabbit polyclonal anti-PKM2 (phosphor-Ser^37^) antibody (PA5-37684) obtained from Thermo Fisher Scientific (Waltham, MA, USA). Mouse monoclonal anti-β-actin antibody (sc-47778), horseradish peroxidase (HRP)-conjugated goat anti-rabbit IgG (sc-2054) and goat anti-mouse IgG (sc-2973) were all purchased from Santa Cruz Biotechnology (Santa Cruz, CA, USA). EGF was purchased from R&D systems (Minneapolis, MN, USA).

### Cell Lines and Cell Culture

The human hepatoma cell lines SNU368, SNU739, Huh-7 and HepG2 were purchased from the Cell Bank of the Chinese Academy of Science (Shanghai, China). All the cells were routinely maintained in RPMI 1640 medium or Dulbecco’s modified Eagle’s medium (DMEM) supplemented with 10% fetal bovine serum (HyClone, UT, USA) at 37 °C in a humidified atmosphere with 5% CO_2_.

### Induction of HCC

Male Sprague-Dawley rats (6-week-old) were purchased from Beijing Weitong Lihua Animal Co. All animal procedures were performed in accordance with the guidelines of the Institutional Animal Care and Use Committee of Henan University (Grant No. HUSOM2018-87). To induce hepatocellular carcinoma, the rats were intraperitoneally injected diethylnitrosamine (DEN) at a dose of 50 mg/kg once a week for 16 weeks. Rats in the control group were intraperitoneally injected with normal saline solution on the same days as the DEN-treated group. At the end of the study, all animals were sacrificed, and the livers were removed. After photographing with a camera, the livers were kept at −80°C for qRT-PCR or Western blotting.

### RNA Extraction and qRT-PCR

mRNA from cancer cells or rat livers were isolated using TRIzol reagent (Invitrogen, Carlsbad, CA, USA) as previous described (Li et al., 2018a). Reverse transcription was performed by using random hexamers and MMLV reverse transcriptase according to the manufacturer’s instructions (Takara, Tokyo, Japan). Relative quantitative real-time PCR was performed in triplicate for each sample using 2×SYBR Green PCR Master Mix (Promega) on an ABI 7500 sequence detection system (Applied Biosystems). Glyceraldehyde phosphate dehydrogenase (GAPDH) was used as an internal control. The relative expression level of the genes was calculated by the 2^−ΔΔCt^ method. Specific primers for human hepatocellular carcinoma cells were as follows: PD-L1, forward: 5′-TGT CAG TGC TAC ACC AAG GC-3′, reverse: 5′-ACA GCT GAA TTG GTC ATC CC-3′. GAPDH, forward: 5′-GAC ACC CAC TCC TCC ACC TTT-3′, reverse: 5′-TTG CTG TAG CCA AAT TCG TTGT-3′. Specific primers for rat were as follows: PD-L1, forward: 5′-TTA TAG TCA CAG CCT GCA GTC ACG -3′, reverse: 5′-ATC GTG ACA TTG CTG CCA TAC TC-3′; GAPDH, forward: 5′-AGC CAT GTA CGT AGC CAT CC-3′, reverse: 5′-GCC ATC TCT TGC TCG AAG TC-3′.

### Western Blot Analysis

Western blot analysis was conducted as previously described (Liu et al., 2018b). Briefly, the total proteins were extracted using ice-chilled RIPA lysis buffer containing 50 mM Tris-HCl (pH 8.0), 150 mM NaCl, 0.5% sodium deoxycholate, 0.1% SDS, 1% NP-40, 5mM EDTA, 0.25 mM PMSF and protease inhibitor cocktail. The nuclear proteins were isolated according to previous report. In brief, the harvested cells were washed three times with cold phosphate buffered saline (PBS) buffer and then resuspended gently in hypotonic buffer containing 20 mM Tris-HCl (pH 7.4), 10 mM NaCl and 3 mM MgCl_2_. After incubation on ice for 15 min, these cells were lysed with a Dounce homogenizer. The homogenate was centrifuged to remove intact cells, followed by centrifugation at 800 g to collect the nuclei. The supernatant contains the cytoplasmic fraction. The nuclear pellets were washed three times with PBS buffer and lysed through sonication. The concentration of total or nuclear protein was determined using a BCA assay kit (Pierce, Rockford, IL). Protein samples were denatured and separated by SDS-polyacrylamide gel electrophoresis (SDS-PAGE). Following separation, the protein was transferred onto a PVDF membrane. The membranes were then blocked with 5% nonfat milk or 3% BSA followed by the incubation of the indicated primary and HRP-conjugated secondary antibodies. The blots were detected using the ECL plus reagents and visualized using a Fluor Chem E Imager (Protein Simple, San Jose, CA, USA).

### ELISA Assay

Supernatants from cancer cell cultures were collected at the indicated time points after EGF stimulation. The content of PD-L1 in the culture medium was measured using ELISA kits (R&D Systems, Minneapolis, MN) according to the instructions provided by the manufacturer. Absorbance was measured at 450 nm by using a Vmax Kinetic microplate reader (Molecular Devices, Sunnyvale, CA).

### Immunofluorescence

The immunofluorescence staining was performed as previously described (Bi et al., 2019). Briefly, Cells grown on coverslips were stimulated with EGF for indicated times and fixed for 10 min in 4% paraformaldehyde at room temperature. After blocked with 5% fetal calf serum for 1 h at room temperature, the cells were stained with the corresponding primary antibody overnight at 4 °C, followed by incubation with Alexa Fluor 488-conjugated secondary antibodies (Invitrogen, Carlsbad, CA, USA) for 1 h at room temperature. Nuclei were labeled with Hoechst 33342 stain for 5 min at room temperature. Images were captured using an LSM510M (Carl Zeiss) confocal microscope.

### Chromatin Immunoprecipitation

Chromatin immunoprecipitation (ChIP) assays were performed using a Simple ChIP plus enzymatic chromatin IP Kit (Cell Signaling Technology) following the manufacturer’s instructions. In brief, cells were cross-linked with 1% formaldehyde for 10 min at 37 °C. Then the chromatin was digested with micrococcal nuclease to generate 150–900 bp DNA/protein fragments. The DNA-protein complexes were immunoprecipitated with the ChIP-grade antibodies and appropriate protein G-agarose beads. Normal rabbit or mouse IgG was used as a negative control. The ChIP samples were verified by qPCR to evaluate histone modification status on the PD-L1 promoter, and the primers were as follows (−1,178 bp to −1,117 bp): forward 5′-GCT GGG CCC AAA CCC TATT - 3′ and reverse 5′-TTT GGC AGG AGC ATG GAG TT-3′.

### Statistical Analysis

Statistical analyses were performed using the GraphPad Prism 8.0 (GraphPad Software, Inc., CA, USA). All data are presented as means ± SEM. The statistical significance of the difference between the two groups was tested by an unpaired, two-tailed Student’s *t*-test. One-way analysis of variance (ANOVA) followed by Tukey or Dunnet's post-tests were used to compare means of multiple experimental groups. *p* < 0.05 was considered to be significant.

## Results

### Activation of EGFR Promotes PD-L1 Transcription in HCC Cells

**FIGURE 1:**
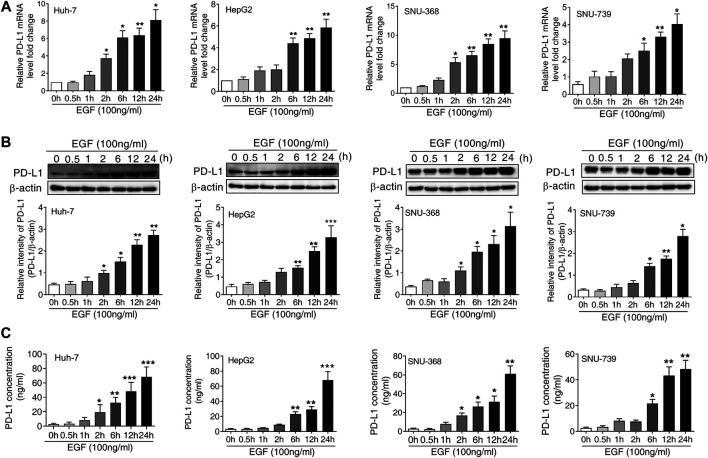
EGF promotes PD-L1 expression in hepatocellular carcinoma cells **(A and B)** A significant increase in PD-L1 mRNA **(A)** and protein expression **(B)** was observed in Huh-7, HepG2, SNU-368 and SNU-739 cells after treated with EGF for indicated time. *p < 0.05, **p < 0.01, ***p < 0.001 compared to control group, one-way ANOVA, *n* = 5 independent experiments per group **(C)** The ELISA assay showed that the concentration of PD-L1 in the cell culture medium was increased after incubation with EGF for indicated time, *p < 0.05, **p < 0.01, ***p < 0.001 compared to control group, one-way ANOVA, *n* = 5 independent experiments per group.

To determine whether the activation of EGFR was responsible for the upregulation of PD-L1 in HCC, we treated Huh-7, HepG 2, SNU-368 and SNU-739 cells with 100 ng/ml EGF and then measured the mRNA and protein expression of PD-L1. Through real time PCR, we found that EGF promoted the mRNA expression of PD-L1 in these cancer cells (Figure 1A). Similarly, incubation of HCC cells with 100 ng/ml EGF also induced a significant increase in PD-L1 protein expression (Figure 1B). Considering that PD-L1 is a secreted protein, we also examined the content of PD-L1 in cell culture medium. As shown in Figure 1C, the level of PD-L1 in cell culture medium increased in EGF-treated group.

**FIGURE 2:**
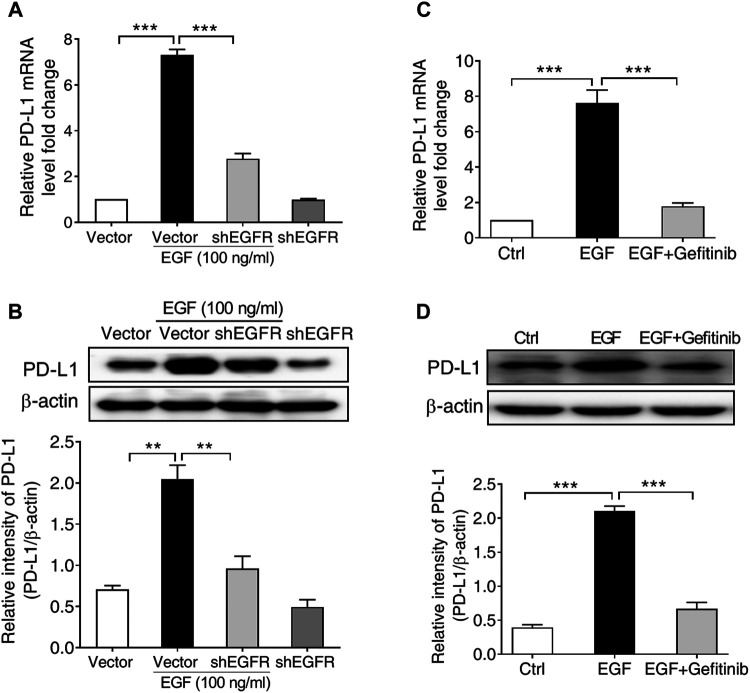
EGFR activation is required for PD-L1 expression in SNU-368 cells. **(A,B)** Knockdown of EGFR with specific shRNA reversed EGF-induced PD-L1 mRNA **(A)** and protein **(B)** expressions in SNU-368 cells. At 24 h post-transfection, the cells were incubated in the presence or absence of 100 ng/ml EGF for 12 h. **p < 0.01, ***p < 0.001, one-way ANOVA, *n* = 4 independent experiments per group. **(C,D)** EGF-induced upregulation of PD-L1 mRNA **(C)** and protein **(D)** was blocked by gefitinib. ***p < 0.001, one-way ANOVA, *n* = 5 independent experiments per group.

Given that EGF-induced PD-L1 mRNA expression in SNU-368 cells was more significant than in other cell lines, we subsequently select SNU-368 cells to explore the underlying mechanism of EGF-induced PD-L1 expression at transcriptional level. In order to determine whether the activation of EGFR contributes to the upregulation of PD-L1 in HCC cells, we first knocked down of EGFR with specific shRNA in SNU-368 cells and evaluated the expression of EGF-mediated PD-L1 expression. Our real time PCR results showed that knockdown of EGFR could block EGF-induced the upregulation of PD-L1 mRNA and protein expression in SNU-368 cells (Figures 2A,B). Then, we examined the effects of Gefitinib, an inhibitor of EGFR tyrosine kinase, on PD-L1 expression in EGF-treated SNU-368 cells. As expected, exposure of SNU-368 cells to 20 µM Gefitinib for 12 h could attenuate the upregulation of PD-L1 mRNA and protein expression induced by EGF (Figures 2C,D). Obviously, EGF can promote the mRNA and protein expression via activation of EGFR in HCC cells.

### Phosphorylation of PKM2 at Ser^37^ Is Required for EGF-Induced PD-L1 Expression

**FIGURE 3:**
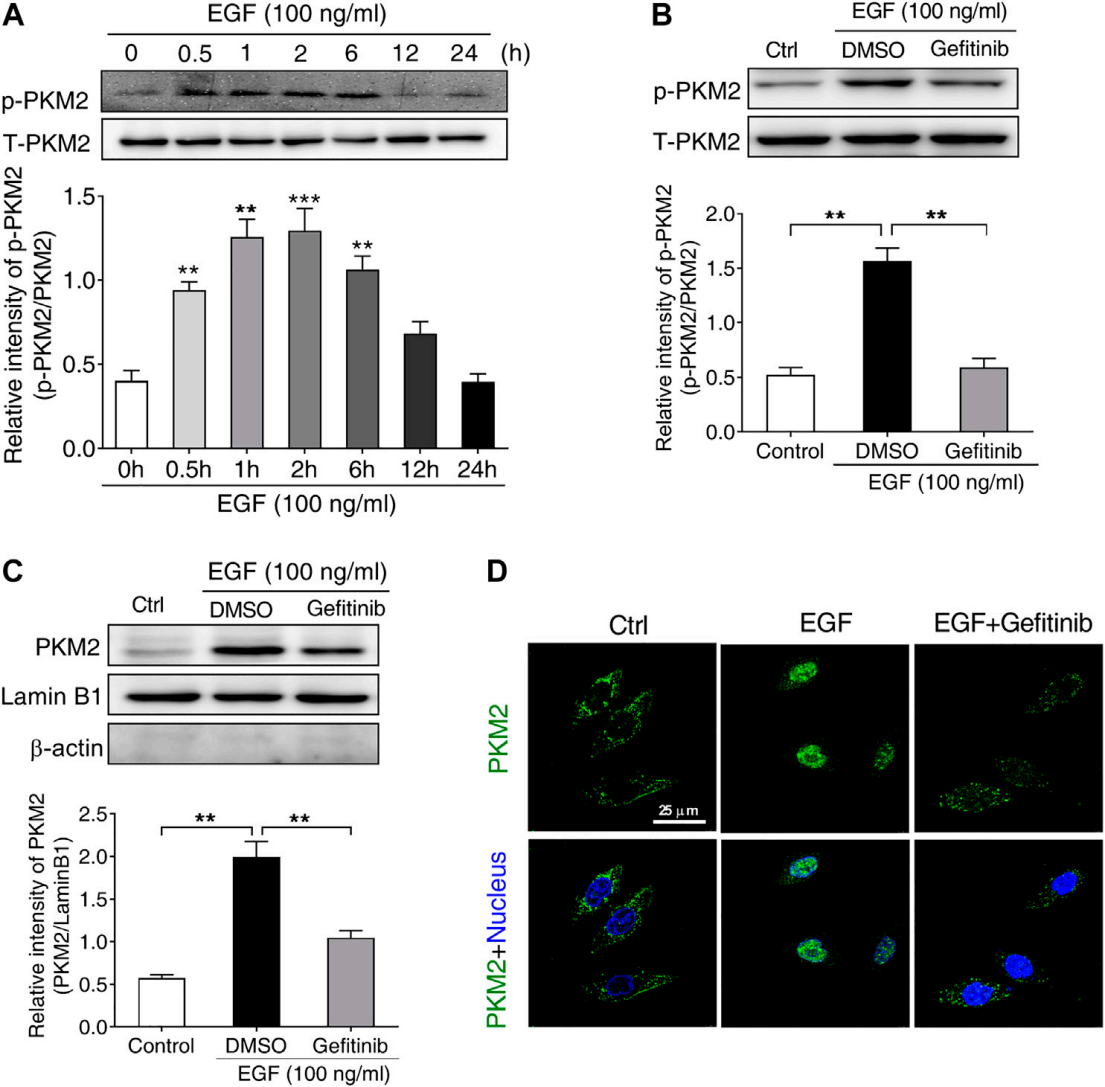
EGF stimulates the phosphorylation and nuclear translocation of PKM2 in SNU-368 cells **(A)** EGF induced an increase in the level of PKM2 Ser^37^ phosphorylation in SNU-368 cells. **p < 0.01, ***p < 0.001, one-way ANOVA, *n* = 6 per group **(B)** Gefitinib attenuated EGF-induced the level of PKM2 Ser^37^ phosphorylation in SNU-368 cells. **p < 0.01, one-way ANOVA, *n* = 5 per group **(C)** EGF-mediated PKM2 nuclear accmulation was reversed by Gefitinib in SNU-368 cells. Lamin B1 was used as internal control and β-actin was used as negative control. **p < 0.01, one-way ANOVA, *n* = 5 per group **(D)** Immunofluorescence staining showed Gefitinib attenuated EGF-induced PKM2 nuclear accmulation in SNU-368 cells.

It has been reported that the phosphorylation of PKM2 at Ser^37^ is required for its nuclear translocation upon EGFR activation, leading to a series of gene expression (Yang et al., 2012b). Based on this report, we supposed that the phosphorylation of PKM2 at Ser^37^ may be involved in EGF-induced PD-L1 expression. To test this hypothesis, we first treated SNU-368 cells with 100 ng/ml EGF and then detected the level of PKM2 Ser^37^ phosphorylation. The results showed that EGF could induce a significant upregulation of PKM2 Ser^37^ phosphorylation in SNU-368 cells (Figure 3A). Then, we asked if EGFR inhibitor Gefitinib could reverse EGF-induced PKM2 Ser^37^ phosphorylation. The results indicated that 20 µM Gefitinib almost completely abolished 100 ng/ml EGF-induced PKM2 Ser^37^ phosphorylation after 2 h coincubation in SNU-368 cells (Figure 3B). Moreover, using a Western blot assay, we also observed increased PKM2 nuclear accumulation in SNU-368 cells after treated with 100 ng/ml for 2 h, while 20 µM Gefitinib blocked 100 ng/ml EGF-induced PKM2 nuclear accumulation (Figure 3C). Immunofluorescence analysis further confirmed these results (Figure 3D).

**FIGURE 4:**
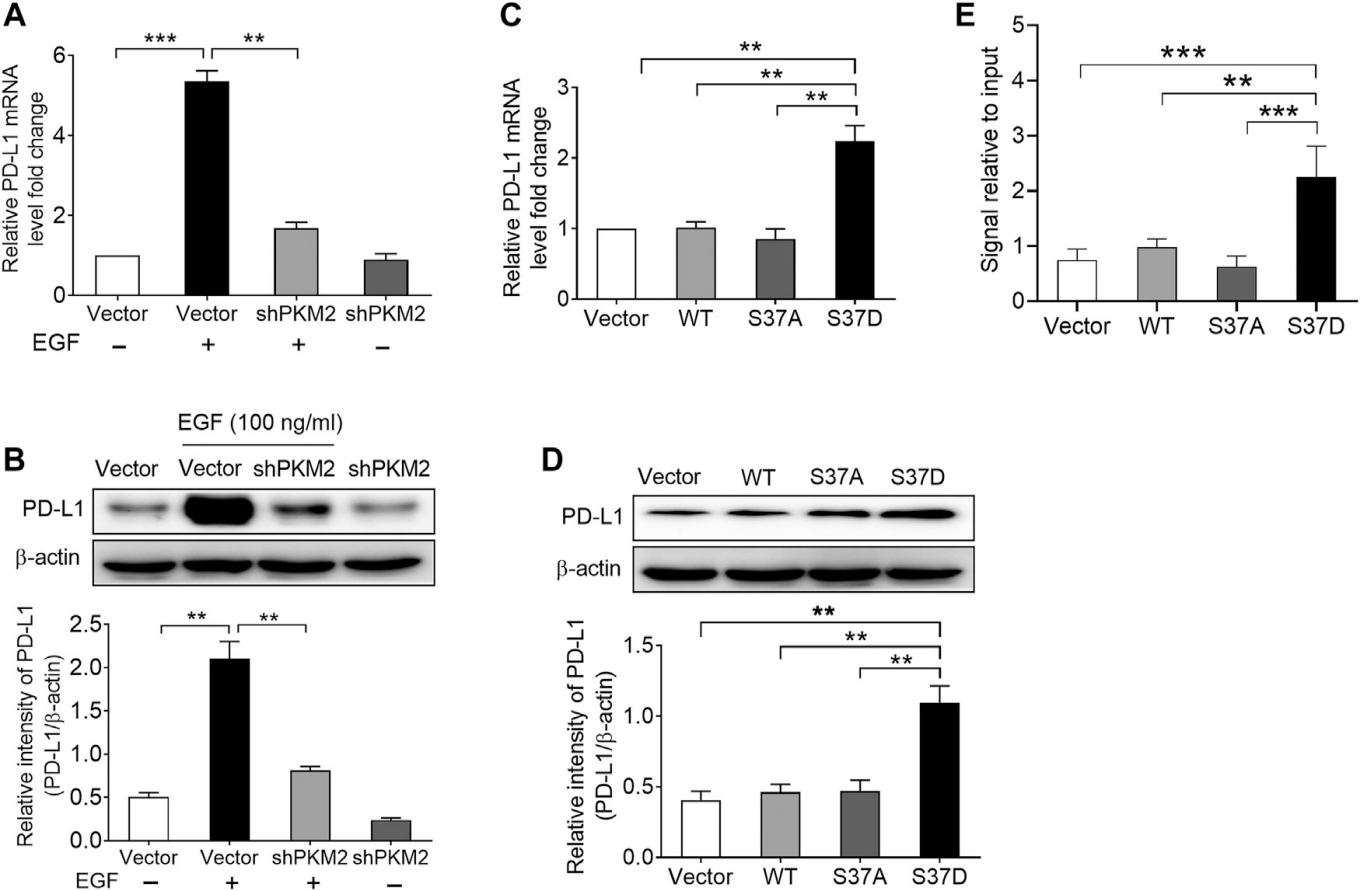
Phosphorylation of PKM2 at Ser37 participates in EGF‐induced PD‐L1 expression. (A,B) PKM2 shRNA blocked EGF‐induced PD‐L1 mRNA **(A)** and protein **(B)** expressions in SNU‐368 cells. At 24 h post‐transfection, the cells were incubated in the presence or absence of 100 ng/ml EGF for 12 h. **p < 0.01, ***p < 0.001, one‐way ANOVA, n = 4 independent experiments per group. (C,D) The expression of a phosphorylation‐mimic PKM2 S37D mutant induced a higher expression of PD‐L1 mRNA **(C)** and protein **(D)** compared with WT PKM2 or the S37A mutant in SNU‐368 cells. **p < 0.01, one-way ANOVA, n = 4 independent experiments per group. **(E)** ChIP analyses showed that the expression of a phosphorylation‐mimic PKM2 S37D mutant resulted in increased binding of PKM2 to PD‐L1 promoter in SNU-368 cells. *p < 0.05, **p < 0.01, one-way ANOVA, n = 5 independent experiments per group.

Subsequently, we examined the effects of PKM2 knockdown on EGF-induced PD-L1 expression in SNU-368 cells. Using real time PCR and Western blot assay, we found that knockdown of PKM2 significantly reversed EGF-induced PD-L1 mRNA and protein expression (Figures 4A,B). Furthermore, we transfected SNU-368 cells with Flag-tagged WT PKM2, a phosphorylation-defective PKM2 S37A mutant, or a phosphorylation-mimic PKM2 S37D mutant and examined the mRNA and protein expression of PD-L1. The results showed that only PKM2 S37D mutant could promote the expression of PD-L1 mRNA and protein in SNU-368 cells (Figures 4C,D). Through chromatin immunoprecipitation (ChIP) analyses, we also demonstrated that only PKM2 S37D mutant, but not WT PKM2 or PKM2 S37A mutant, could induce an increased PKM2 binding to PD-L1 promoter in SNU-368 cells (Figure 4E). Thus, phosphorylation of PKM2 at Ser^37^ is essential for EGF-induced PD-L1 expression in HCC cells.

### PKM2-Induced Phosphorylation of Histone H3 is Involved in EGF-Mediated PD-L1 Expression in HCC Cells

**FIGURE 5:**
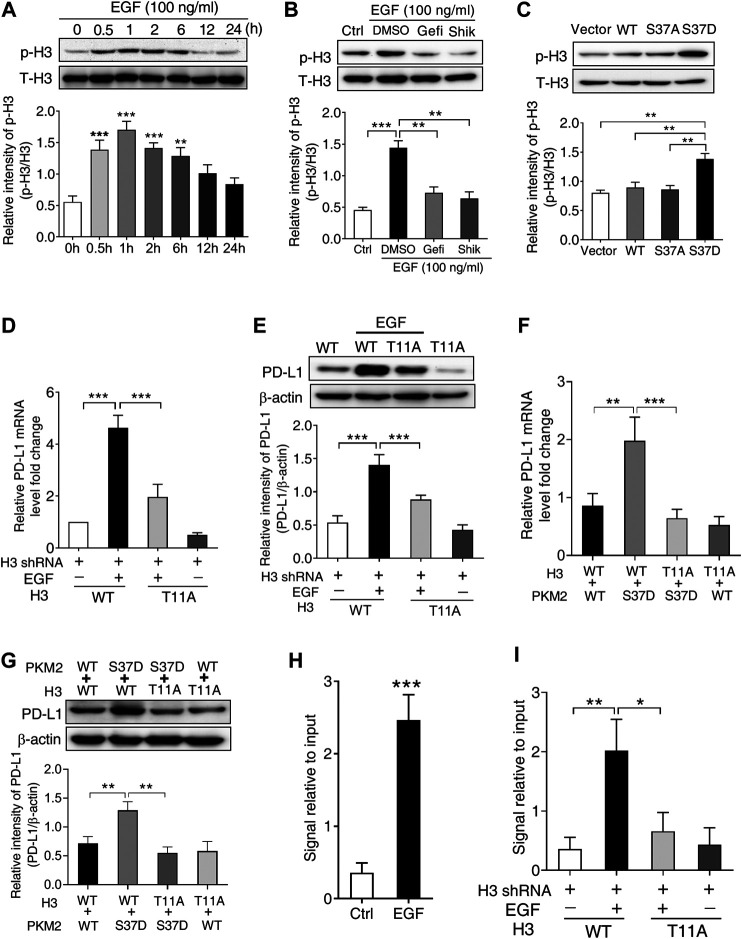
PKM2-induced phosphorylation of histone H3 at Thr^11^ is required for EGF-mediated PD-L1 transcription in SNU-368 cells. **(A)** EGF treatment induced a significant increase in H3 Thr^11^ phosphorylation in SNU-368 cells. **p < 0.01, ***p < 0.001, one-way ANOVA, *n* = 5 independent experiments per group, **(B)** EGF-induced H3-Thr^11^ phosphorylation was attenuated by Gefitinib and shikonin after 1 h co-incubation in SNU-368 cells, **p < 0.01, ***p < 0.001, one-way ANOVA, *n* = 5 independent experiments per group, **(C)** expression of the phosphorylation-mimic PKM2 S37D mutant increased H3-Thr^11^ phosphorylation compared with the WT PKM2 or the S37A mutant in SNU-368 cells. **p < 0.01, one-way ANOVA, *n* = 5 independent experiments per group, **(D and E)** Reconstituted expression of RNAi-resistant histone H3-T11A abrogated EGF-induced the mRNA, **(D)**, and protein **(E)** expression of PD-L1 in endogenous H3-depleted SNU-368 cells. At 24 h post-transfection, cells were incubated in the presence or absence of 100 ng/ml EGF for 12 h ***p < 0.001, one-way ANOVA, *n* = 5 independent experiments per group **(F and G)** Reconstituted expression of WT histone H3 and PKM2 S37D could induce PD-L1 mRNA **(F)** and protein **(G)** expression which could be blocked when WT histone H3 was replaced with histone H3 T11 A mutant in endogenous histone H3-depleted SNU-368 cells. **p < 0.01, ***p < 0.001, one-way ANOVA, *n* = 5 independent experiments per group **(H)** ChIP analysis showed that EGF treatment for 6 h resulted in enhanced H3-Thr^11^ phosphorylation at the PD-L1 promoter in SNU-368 cells. **p < 0.01, a two-tailed unpaired *t*-test, *n* = 5 independent experiments per group **(I)** ChIP analysis showed reconstituted expression of histone H3 T11A mutant abrogated EGF-induced enhanced H3-Thr^11^ phosphorylation at the PD-L1 promoter in endogenous histone H3-depleted SNU-368 cells.

PKM2 has been reported to phosphorylate histone H3 at Thr^11^ site in response to EGF stimulation, which is required for subsequent gene expression (Yang et al., 2012a). Therefore, we first treated SNU-368 cells with 100 ng/ml EGF and examined the level of histone H3 Thr^11^ phosphorylation. The results indicated that EGF treatment increased histone H3 Thr^11^ phosphorylation in SNU-368 cells (Figure 5A). To investigate whether EGF-induced H3 Thr^11^ phosphorylation depended on PKM2, we then examined the effects of EGFR inhibitor Gefitinib and PKM2 inhibitor shikonin on EGF-induced H3 Thr^11^ phosphorylation. Through Western blot assay, we discovered that both Gefitinib (20 µM) and Shikonin (5 µM) could block EGF-mediated the upregulation of histone H3 Thr^11^ phosphorylation (Figure 5B). To test whether the phosphorylation of PKM2 at Ser^37^ is instrumental for histone H3 Thr^11^ phosphorylation, we expressed a Flag-tagged WT PKM2, a phosphorylation-defective PKM2 S37A mutant or a phosphorylation-mimic PKM2 S37D mutant in SNU-368 cells and measured the level of histone H3-Thr^11^ phosphorylation. The results indicated that the phosphorylation-mimic PKM2 S37D mutant induced a higher level of histone H3 Thr^11^ phosphorylation compared with WT PKM2 and PKM2 S37A mutant (Figure 5C). These results suggested that phosphorylation of PKM2 at Ser^37^ is required to EGF-induced H3-Thr^11^ phosphorylation.

To investigate whether H3-Thr^11^ phosphorylation was involved in EGF-induced PD-L1 expression, we expressed RNAi-resistant WT histone H3 or histone H3-T11A in endogenous histone H3-depleted SNU-368 cells, the results showed that mutation of Thr^11^ of histone H3 into alanine (Ala, A) abrogated EGF-induced mRNA and protein expression of PD-L1 (Figures 5D,E). To strengthen the evidence for the causal relationship of PKM2/H3 T11 phosphorylation on PD-L1 expression, we then explored the effect of H3 T11A mutation on the PKM2 S37D overexpression-induced PD-L1 expression in endogenous histone H3-depleted SNU-368 cells. Through real time PCR and Western blot, we found reconstituted expression of WT histone H3 and PKM2 S37D could induce PD-L1 mRNA and protein expression in H3-depleted SNU-368 cells (Figures 5F,G), however, these increased expression of PD-L1 mRNA and protein could be blocked when WT histone H3 was replaced with histone H3 T11A mutant in endogenous histone H3-depleted SNU-368 cells (Figures 5F,G). ChIP assay also suggested that EGF treatment resulted in enhanced H3-Thr^11^ phosphorylation at the PD-L1 promoter (Figure 5H), while mutation of Thr^11^ of histone H3 into alanine (Ala, A) abrogated EGF-induced enhanced H3-Thr^11^ phosphorylation at the PD-L1 promoter in endogenous histone H3-depleted SNU-368 cells (Figure 5I). These data confirmed our hypothesis that PKM2-induced H3-Thr^11^ phosphorylation is involved in EGF-mediated PD-L1 expression in HCC cells.

**FIGURE 6:**
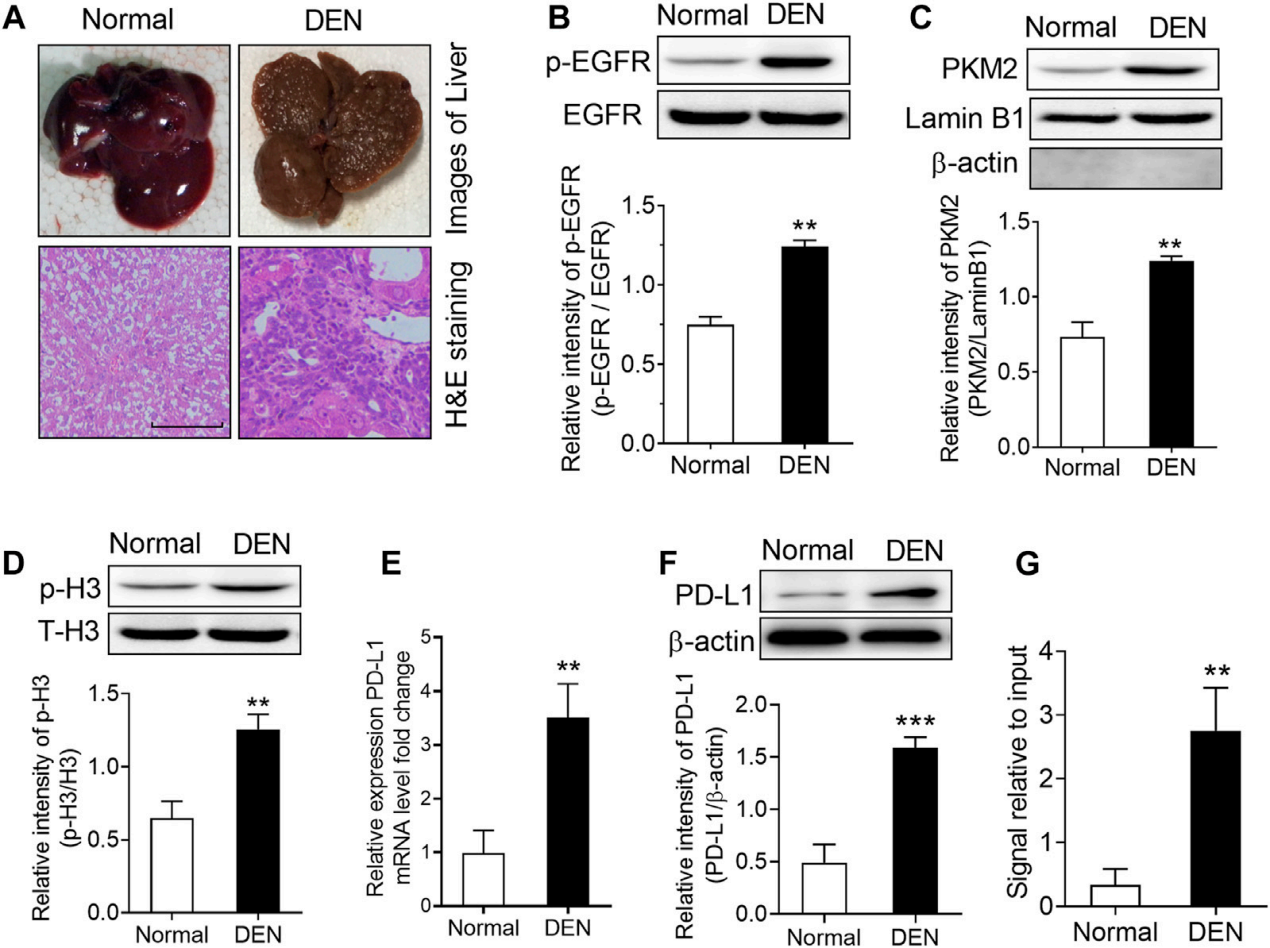
DEN treatment induced a significant upregulation of phospho-EGFR, phospho-H3, and PKM2 nuclear accumulation in rat livers. **(A)** Top, representative photos of livers from normal and DEN-treated rats; bottom, representative images of H&E-stained livers. **(B)** The phosphorylational level of EGFR at Tyr^1068^ was increased in the livers of DEN-treated rats. **p < 0.01, two-tailed unpaired t-test, *n* = 8 rats per group. **(C)** The expression of PKM2 nuclear protein was upregulated in the livers of DEN-treated rats. Lamin B1 was used as an internal control, and β-actin was used as a negative control. **p < 0.01, two-tailed unpaired *t*-test, *n* = 8 rats per group. **(D)** The phosphorylational level of H3-Thr^11^ was increased in the livers of DEN-treated rats. **p < 0.01, two-tailed unpaired *t*-test, *n* = 8 rats per group. **(E,F)** The expression of PD-L1 mRNA **(E)** and protein **(F)** was increased in the livers of DEN-treated rats. **p < 0.01, ***p < 0.001, two-tailed unpaired t-test, *n* = 8 rats per group. **(G)** ChIP analyses showed that DEN administration resulted in enhanced H3-Thr^11^ phosphorylation at the PD-L1 promoter in rats. **p < 0.01, two-tailed unpaired *t*-test, *n* = 8 rats per group.

In order to expand our findings *in vivo*, we subsequently injected diethylnitrosamine (DEN) into rats to set up a model of hepatocellular carcinoma. As the results shown in Figure 6A, numerous tumor nodules occurred in the livers of rats that received DEN administration for 16 weeks. Then, we measured the level of EGFR phosphorylation, the expression PKM2 nuclear protein and H3-Thr^11^ phosphorylation in these rats. The results showed that DEN treatment could induce a significant increase in EGFR phosphorylation, PKM2 nuclear protein and H3-Thr^11^phosphorylationin in the rat livers (Figures 6B–D). Moreover, we also found that the mRNA and protein expression of PD-L1 was increased in the livers of DEN-treated rats (Figures 6E,F). Meanwhile, a ChIP assay also demonstrated that DEN administration resulted in enhanced H3-Thr^11^ phosphorylation at the PD-L1 promoter in rats (Figure 6G). Taken together, these *in vivo* results also implied that PKM2-induced H3-Thr^11^ phosphorylation is involved in EGF-mediated PD-L1 expression in HCC.

## Discussion

High expression of PD-L1 is significantly associated with tumor aggressiveness and poor prognosis in HCC patients who were never treated via immunotherapy (Liao et al., 2019; Mocan et al., 2019). During tumor development and growth, PD-L1 can enhance immune evasion by suppression of recruitment and activation of T cells in tumor microenvironment (Macek Jilkova et al., 2019). Recently, emerging evidence come to explore the underlying mechanism of PD-L1 expression in HCC. For example, osteopontin (OPN) promotes the PD-L1 expression in HCC via activation of the colony stimulating factor-1 (CSF1)-CSF1 receptor pathway in macrophages (Zhu et al., 2019). Knockdown of MYC expression can induce PD-L1 expression both at mRNA and protein levels (Zou et al., 2018). A key glycolytic enzyme, PFKFB3, mediated the increased expression of PD-L1 via activating the nuclear factor kappa B signaling pathway in HCC (Chen et al., 2019a). Although accumulating evidences suggested the activation of EGFR contributes to PD-L1 expression in several cancers (Li et al., 2018b; Chen et al., 2019b), it is still unknown whether EGF/EGFR signal was involved in PD-L1 expression in HCC. In the present study, we also observed that EGF could induce PD-L1 expression at the transcriptional level in HCC cells, while inhibition of EGFR dramatically attenuated EGF-induced PD-L1 expression. Therefore, we supposed that EGF/EGFR signal may be critical in regulating PD-L1 expression in many cancers. However, because we used Gefitinib to block the activity of EGFR tyrosine kinase, this may bring a risk of off targets effects due to the problem of dosage and specificity. Indeed, this is a limitation of our study.

Overexpression of PKM2 has been observed in a variety of malignant tumors, which is related to the tumor proliferation, progression and drug resistant (Wong et al., 2015; Bhardwaj and Das, 2016; Li et al., 2018c). As a glycolytic enzyme, PKM2 normally located in the cytoplasm functions as a prominent driver of the Warburg effect that plays a dominant role in cancer metabolism (Wong et al., 2015; Wang et al., 2017). In addition, PMK2 has nonmetabolic functions in malignant cells through acting as a glycolytic enzyme or transcriptional coactivator. For example, PKM2 could translocate to the nucleus and form a complex with HIF-1α subunit to promote the transcription of HIF-1α targeted genes (Azoitei et al., 2016). Nuclear dimeric PKM2 also directly phosphorylates signal transducer and activator of transcription 3 (STAT3) at Tyr^705^, resulting in aggressive progression of colorectal cancer (Yang et al., 2014). Here, our results also suggested that nuclear PKM2 could serve as a transcriptional cofactor to induce PD-L1 transcription in response to EGF stimulation in hepatocellular carcinoma cells. Accumulating evidences illuminated that post-translational modifications including phosphorylation and acetylation are critical for the protein localization and functional modulation of PKM2(Yang et al., 2012b; Wang et al., 2017). For instance, the Ser^202^ phosphorylation of PKM2 by AKT1 is essential for the nuclear localization of PKM2 protein under IGF-1 stimulation (Park et al., 2016). Acetyltransferase p300 could acetylate PKM2 at Lys^433^, leading to the accumulation PKM2 in the nucleus and the increases of both tyrosine and threonine kinase activities (Lv et al., 2013). Especially, Yang et al. (2012b) have found that phosphorylation of PKM2 at Ser^37^ by ERK1/2 promotes the translocation of PKM2 to the nucleus upon EGFR activation. Consistent with Yang report, we also demonstrated that Ser^37^-phosphorylated PKM2 is vital for PKM2 nucleus localization in HCC. Meanwhile, we believe ERK1/2 may be also responsible for the phosphorylation of PKM2 at Ser^37^ in HCC cells.

Histone H3 phosphorylation has been regarded as one of the most frequent epigenetic modifications that affects chromatin structure and gene transcription (Cerutti and Casas-Mollano, 2009). For example, phosphorylation of histone H3 at Ser^10^ was required for AP-1 activation in nasopharyngeal carcinoma (Li et al., 2013); phosphorylation H3 Thr^45^ by Akt has been reported to facilitate transcriptional termination in response to DNA damage (Hurd et al., 2009); histone H3-Thr^11^ phosphorylation induced by PKM2 is required for cyclin D1 and c-Myc expression under EGF stimulation (Yang et al., 2012a). In support of these observations, our results suggested that PKM2-mediated histone H3-Thr^11^ phosphorylation participated in EGF-induced PD-L1 transcriptional expression. Interestingly, several studies suggested that histone H3 acetylation also play a key role in the regulation of PD-L1 gene expression in cancer cells (Lei et al., 2015; Wang et al., 2020). Given that the process of H3 phosphorylation usually recruits a histone modifying enzyme that would in turn generate the second modification (Cheung et al., 2000; Yang et al., 2012a), we supposed histone H3 acetylation may be also involved in EGF-induced PD-L1 expression in HCC cells. In line with this opinion, Yang et al. (2012a) found that H3-Thr^11^ phosphorylation induced by EGF is required for histone deacetylase 3 (HDAC3) disassociation from some gene promoters and subsequent acetylation of histone H3 at Lys^9^. Notably, a recent study suggested that enhancer of zeste homolog 2 (EZH2) can suppress PD-L1 expression by directly upregulating H3K27me3 levels at CD274 (encoding PD-L1) promoter regions in hepatoma cells (Xiao et al., 2019). Combined with Dong et al. (2018) findings that EGF can promote EZH2 expression in human lens epithelial cells (HLECs), therefore, we cannot exclude that EGF can regulate PD-L1 expression through inducing EZH2 overexpression in HCC.

**FIGURE 7:**
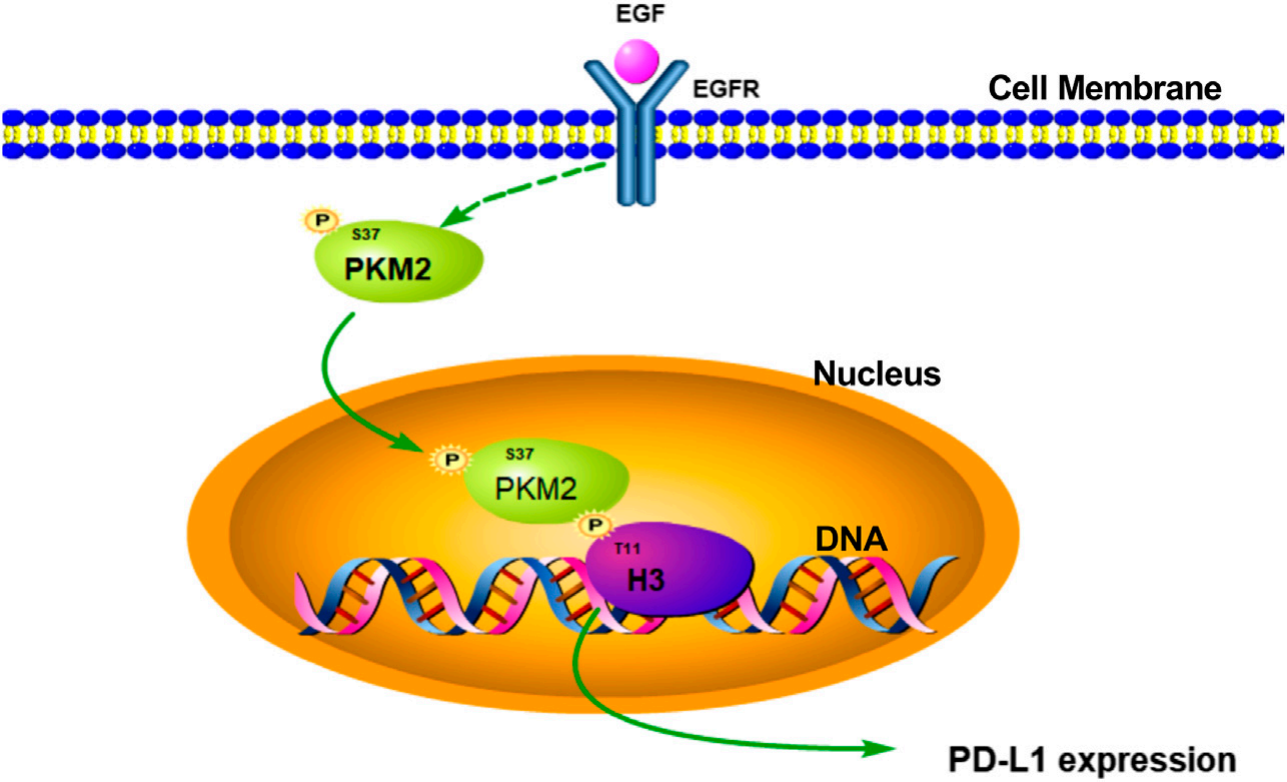
Schematic representation of the activation of EGF induced PD-L1 transcription in hepatocellular carcinoma cells. EGF stimulation induced PKM2 phosphorylation at Ser^37^ site. Then, the phosphorylated PKM2 translocates into the nucleus to phosphorylate histone H3 at Thr^11^, leading to PD-L1 transcription in HCC.

In conclusion, our study firstly suggested that EGF could induce PKM2 phosphorylation at Ser^37^ and translocation of the PKM2 protein to the nucleus, the nuclear PKM2 then phosphorylates histone H3 at Thr^11^ and the subsequent expression of PD-L1 in HCC (Figure 7). Furthermore, these results also revealed that targeting PKM2 or histone H3-Thr^11^ may be an effective treatment approach for HCC in the future.

## Data Availability

The raw data supporting the conclusions of this article will be made available by the authors, without undue reservation.
