# Corrigendum: PKM2-Induced the Phosphorylation of Histone H3 Contributes to EGF-Mediated PD-L1 Transcription in HCC

**DOI:** 10.3389/fphar.2021.724799

**Published:** 2021-07-15

**Authors:** Xiao Wang, Chao Liang, Xin Yao, Ruo-Han Yang, Zhan-Sheng Zhang, Fan-Ye Liu, Wen-Qi Li, Shu-Hua Pei, Jing Ma, Song-Qiang Xie, Dong Fang

**Affiliations:** ^1^ Institute for Innovative Drug Design and Evaluation, School of Pharmacy, Henan University, Kaifeng, China; ^2^ Institute of Chemical Biology, School of Pharmacy, Henan University, Kaifeng, China

**Keywords:** epidermal growth factor, pyruvate kinase isoform M2, histone H3, programmed death-ligand-1, hepatocellular carcinoma

In the original article, there were several errors in “**Figures and Figure legends**.”

In Figure 2 as published, the letters B and C which indicated the figure order were marked in reverse. In Figure 4, the letters from B to E which indicated the figure order were marked in reverse, and the figure label “EGF” was missed in the second and third bands in western blots in Figure 4B. In Figure 6B, the figure label “EGFR” was marked as “β-actin” mistakenly. Besides, we mistakenly wrote PD-L1 as DKK1 in Figure legends 2, 4, and 6 because of our carelessness. The corrected Figures 2, 4 and 6 appear below.

**FIGURE 2:**
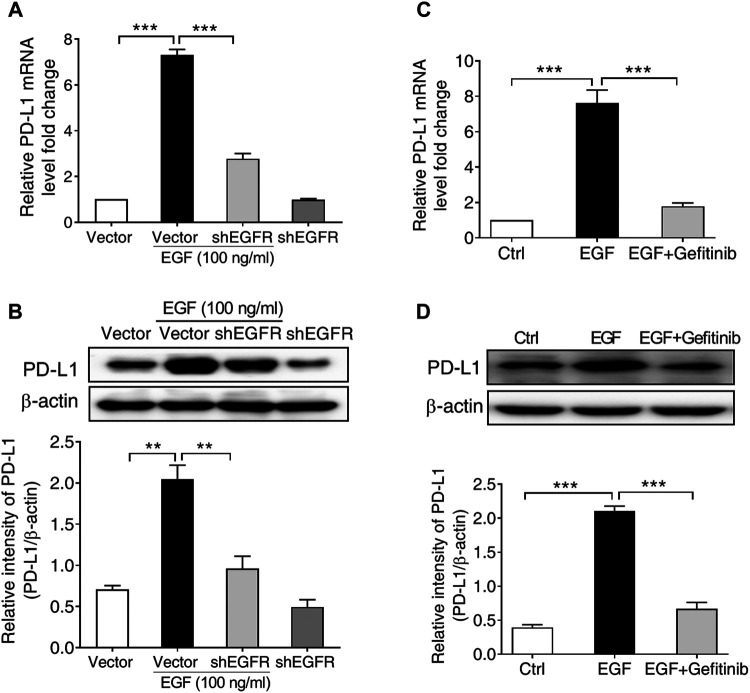
EGFR activation is required for PD-L1 expression in SNU-368 cells. **(A,B)** Knockdown of EGFR with specific shRNA reversed EGF-induced PD-L1 mRNA **(A)** and protein **(B)** expressions in SNU-368 cells. At 24 h post-transfection, the cells were incubated in the presence or absence of 100 ng/ml EGF for 12 h. **p < 0.01, ***p < 0.001, one-way ANOVA, *n* = 4 independent experiments per group. **(C,D)** EGF-induced upregulation of PD-L1 mRNA **(C)** and protein **(D)** was blocked by gefitinib. ***p < 0.001, one-way ANOVA, *n* = 5 independent experiments per group.

**FIGURE 4:**
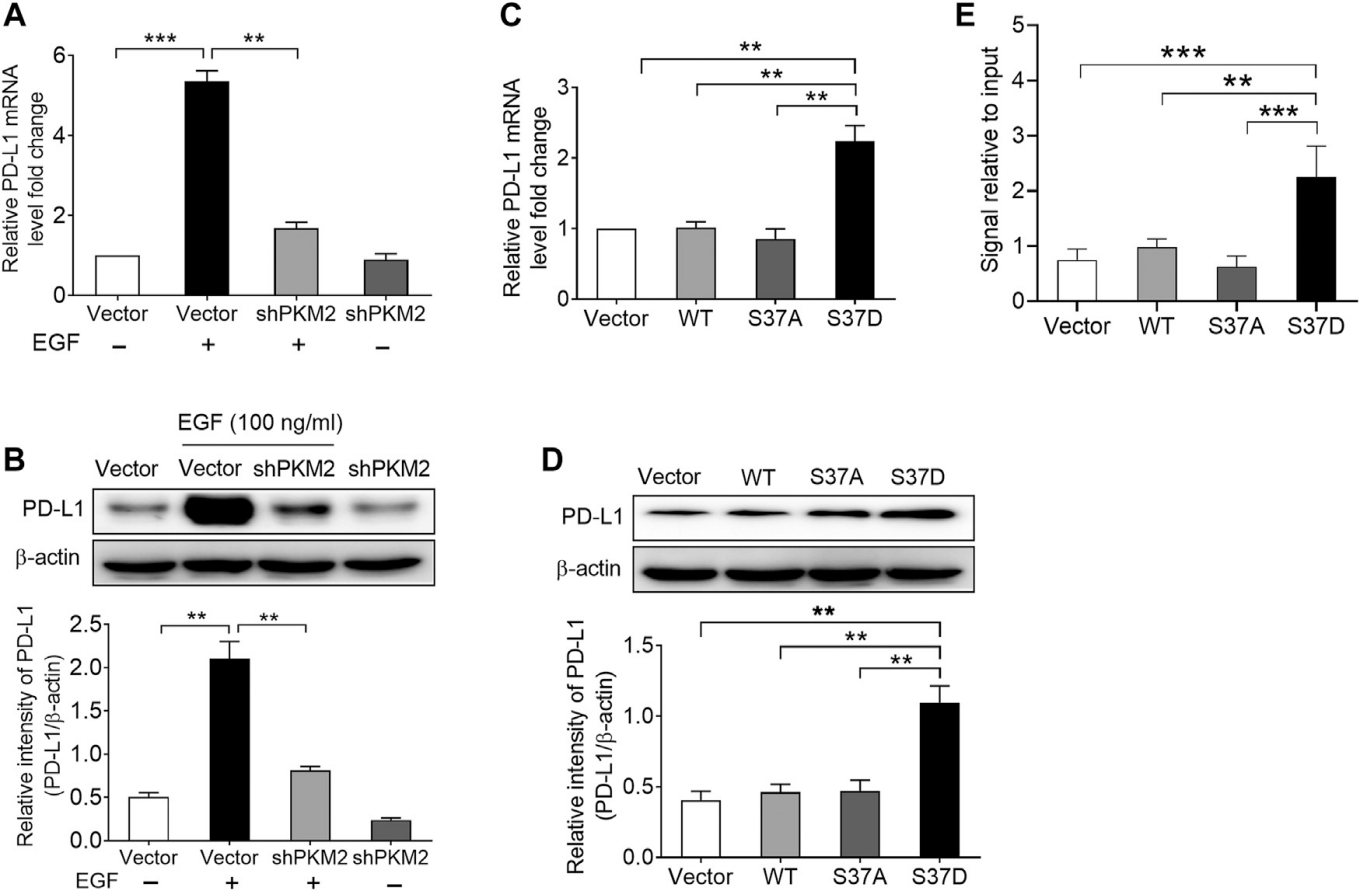
Phosphorylation of PKM2 at Ser37 participates in EGF‐induced PD‐L1 expression. (A,B) PKM2 shRNA blocked EGF‐induced PD‐L1 mRNA **(A)** and protein **(B)** expressions in SNU‐368 cells. At 24 h post‐transfection, the cells were incubated in the presence or absence of 100 ng/ml EGF for 12 h. **p < 0.01, ***p < 0.001, one‐way ANOVA, n = 4 independent experiments per group. (C,D) The expression of a phosphorylation‐mimic PKM2 S37D mutant induced a higher expression of PD‐L1 mRNA **(C)** and protein **(D)** compared with WT PKM2 or the S37A mutant in SNU‐368 cells. **p < 0.01, one-way ANOVA, n = 4 independent experiments per group. **(E)** ChIP analyses showed that the expression of a phosphorylation‐mimic PKM2 S37D mutant resulted in increased binding of PKM2 to PD‐L1 promoter in SNU-368 cells. *p < 0.05, **p < 0.01, one-way ANOVA, n = 5 independent experiments per group.

**FIGURE 6:**
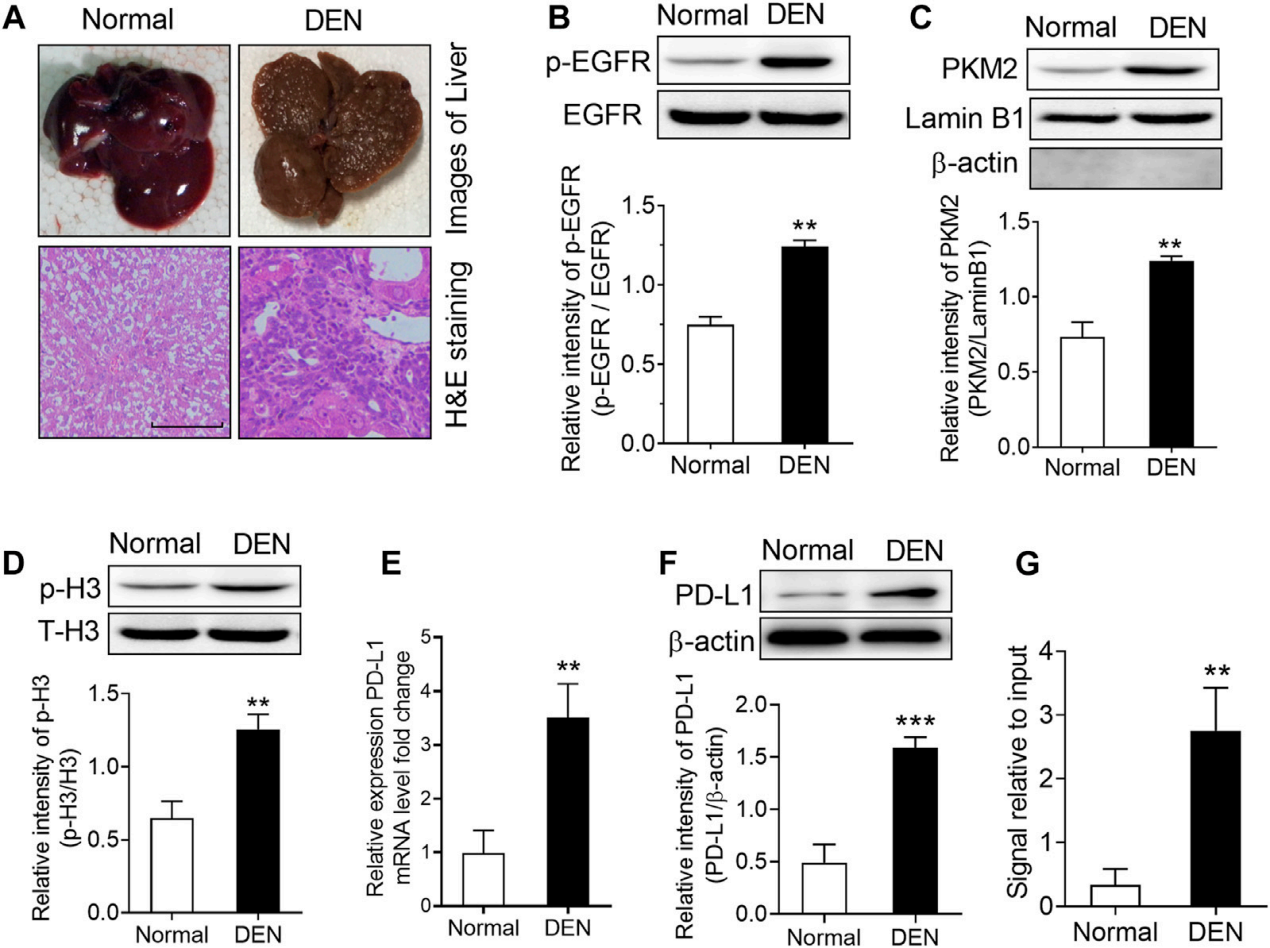
DEN treatment induced a significant upregulation of phospho-EGFR, phospho-H3, and PKM2 nuclear accumulation in rat livers. **(A)** Top, representative photos of livers from normal and DEN-treated rats; bottom, representative images of H&E-stained livers. **(B)** The phosphorylational level of EGFR at Tyr^1068^ was increased in the livers of DEN-treated rats. **p < 0.01, two-tailed unpaired t-test, *n* = 8 rats per group. **(C)** The expression of PKM2 nuclear protein was upregulated in the livers of DEN-treated rats. Lamin B1 was used as an internal control, and β-actin was used as a negative control. **p < 0.01, two-tailed unpaired *t*-test, *n* = 8 rats per group. **(D)** The phosphorylational level of H3-Thr^11^ was increased in the livers of DEN-treated rats. **p < 0.01, two-tailed unpaired *t*-test, *n* = 8 rats per group. **(E,F)** The expression of PD-L1 mRNA **(E)** and protein **(F)** was increased in the livers of DEN-treated rats. **p < 0.01, ***p < 0.001, two-tailed unpaired t-test, *n* = 8 rats per group. **(G)** ChIP analyses showed that DEN administration resulted in enhanced H3-Thr^11^ phosphorylation at the PD-L1 promoter in rats. **p < 0.01, two-tailed unpaired *t*-test, *n* = 8 rats per group.

The authors apologize for this error and state that this does not change the scientific conclusions of the article in any way. The original article has been updated.

